# Bioadhesive Nanoparticles in Topical Drug Delivery: Advances, Applications, and Potential for Skin Disorder Treatments

**DOI:** 10.3390/pharmaceutics17020229

**Published:** 2025-02-10

**Authors:** Rashed M. Almuqbil, Bandar Aldhubiab

**Affiliations:** Department of Pharmaceutical Sciences, College of Clinical Pharmacy, King Faisal University, Al-Ahsa 31982, Saudi Arabia; baldhubiab@kfu.edu.sa

**Keywords:** bioadhesives, nanoparticles, skin disorders, targeted drug delivery, prolonged retention time, deeper drug penetration

## Abstract

Skin disorders are the fourth most common cause of all diseases, which affect nearly one-third of the world’s population. Topical drug delivery can be effective in treating a range of skin disorders, including microbial infections, skin cancer, dermatitis, burn injury, wounds, and psoriasis. Bioadhesive nanoparticles (BNPs) can serve as an efficient topical drug delivery system as they can serve dual purposes as bioadhesives and nanocarriers, which can mediate targeted drug delivery, prolong retention time, and deepen drug penetration through skin layers. There is an increasing demand for BNP-based applications in medicine because of their various advantages, including biodegradability, flexibility, biocompatibility, and enhanced adhesive strength. A number of BNPs have already been developed and evaluated as potential topical drug delivery systems. In addition, a range of studies have already been carried out to evaluate the potential of BNPs in the treatment of various skin disorders, including atopic dermatitis, irritant contact dermatitis, skin cancer, psoriasis, microbial infections, wounds, and severe burn injuries. This review article is timely and unique, because it provides an extensive and unique summary of the recent advances of BNPs in the treatment of wide-ranging skin disorders. Moreover, this review also provides a useful discussion on the bioadhesion mechanism and various biopolymers that can be used to prepare BNPs.

## 1. Introduction

Drug delivery through the skin can avoid numerous drawbacks associated with parenteral, inhalation, and oral routes [1]. Skin disorders affect nearly one-third of the world’s population. In humans, skin disorder is the fourth most common cause of all diseases. In spite of this substantial disease burden, the impact of skin disorders is often underestimated [2,3]. Topical drug delivery is an important issue in treating a range of skin disorders including microbial infections, skin cancer, dermatitis, burn injury, wounds, and psoriasis [4,5].

Adhesion is defined as the tendency of similar or different particles or surfaces to cling to one another. Certain mechanical activities and intermolecular forces are the forces accountable for adhesion. Adhesives are substances that are able to hold or attach materials together in a functional manner that opposes separation. Adhesives are extensively used in numerous industries, including biomedicine, histopathology, interior design, aircraft, food packaging, and many more [6]. In terms of biomedical applications, bioadhesives are commonly used as adhesives in the replacement of sutures and staples for closing wounds, because traditional sutures and staples have several drawbacks including poor skin healing, keloid formation, infection, and scarring [7]. Furthermore, bioadhesives are easy to use, reduce the risk of bleeding, substantially reduce the duration of surgery, and markedly decrease healthcare costs. Bioadhesives are predominantly natural polymers that might include various substances, predominantly carbohydrates and proteins. Carbohydrates, including starch, and proteins, including gelatin, have been used as general-purpose glues for numerous years. Because of their biocompatibility, there is a growing interest in bioadhesives because of their many uses in biomedical applications, including skin or other body tissues [8,9]. Researchers have already developed several bioadhesives to use as potential drug delivery systems [10].

An ideal bioadhesive should have a number of properties. It should have high chain flexibility, high molecular weight, a sufficient amount of hydrogen-bonding chemical groups, be excreted in an unaltered form, or should biologically be degraded to a nontoxic and inactive form [6]. Moreover, ideal bioadhesives must swell in the aqueous biological environment of the site of absorption, should have high drug-loading capacity, and should have the capacity for the controlled release of the active ingredient when swollen [11]. In the human body, different bioadhesives are required for different purposes for diversity in the complex cellular systems and variations in mechanical features of tissues. Nonetheless, all bioadhesives ought to contain basic properties for clinical uses. Bioadhesive-associated important factors that need to be considered for clinical applications include easy application, biodegradation within a time period, enhanced healing, promoting new tissue formation, not imparting toxic effects, and being cheap and easily producible [6].

**Figure 1:**
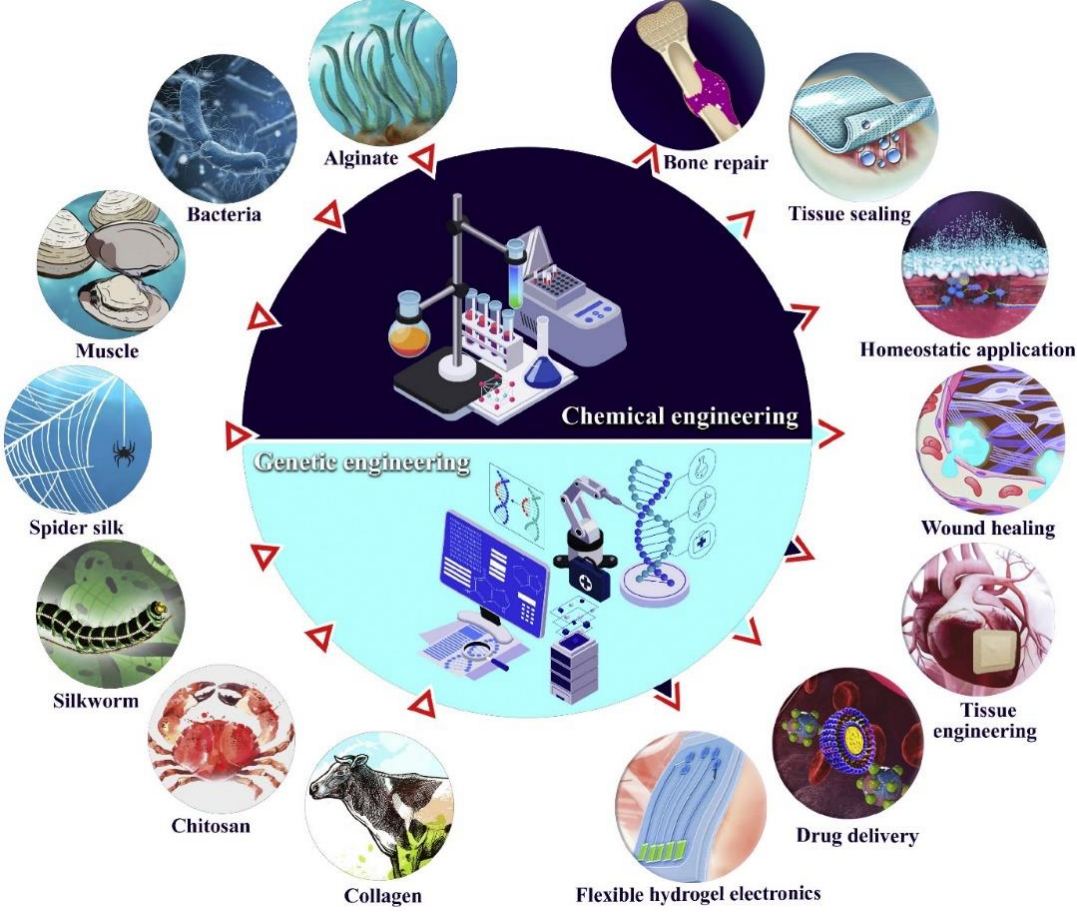
Bioadhesives with wide-ranging biomedical applications. Reproduced with permission from Elsevier, Reference [15].

Nanoparticles (NPs) are small particles that range between 1 and 100 nm in size. NPs are widely used in rapidly emerging sectors including nanomedicine and nanodelivery systems. Typically, NPs are colloidal particles of drugs that might be adsorbed, encapsulated, or entrapped. A drug molecule must reach the target site of action to exert its optimum effect. NPs offer several advantages when added in a formulation, including increased drug safety and efficacy, the modulation of the pharmacokinetics of drug release, and targeting drugs to a specific site [12,13]. Several NPs with bioadhesive properties have already been identified as potential drug carriers, as they can effectively penetrate and target the site of action. There is a growing demand for developing bioadhesive NPs (BNPs) because of the benefits of small particle size and increased surface area. Thus, BNPs have an enormous potential as potential carriers for therapeutics in topical drug delivery systems. Various studies have explored the efficacy of natural and synthetic biopolymer-based BNPs for local drug delivery. Moreover, bioadhesives can be genetically and chemically modified to enhance their functionalities and uses (Figure 1). It was observed that BNPs are effective in inducing particle uptake and prolonging the drug retention time owing to their highly specific surface area and small size [14]

This review provides an in-depth summary of the recent advances of BNPs in terms of the treatment of various skin disorders. Moreover, this review also provides a useful discussion on the bioadhesion mechanism and various biopolymers that can be used to prepare BNPs.

## 2. Search Strategies

A range of very well-known databases, including PubMed, Scopus, Cochrane Library, Scopus, ScienceDirect, Web of Science, and Google Scholar, have been searched. These databases were searched up to January 2025 by using terms or combination of terms including bioadhesive nanoparticles, skin, topical drug delivery, applications, skin disorders, and treatments. Following a systematic search of the databases, the articles were manually screened, and the articles that met the inclusion criteria were further fully reviewed and sources of the selected articles were cited wherever used.

## 3. Mechanism of Bioadhesion

Bioadhesion is a process in which a natural, semi-synthetic, or synthetic macromolecule attaches with the mucus or epithelial surface for an extended period of time. Interfacial forces largely govern the bond between two materials. Bioadhesion shows similarity to the typical adhesion process; however, the major difference is that bioadhesion includes distinct features of biological surfaces and organisms [16]. Bioadhesion can be grouped into non-specific and specific bioadhesion [17]. Non-specific bioadhesives (for example- carbomers, carbopol, chitosan, and polycarbophil) have the capacity to bind with both the mucosal layer and cell surface [18]. On the other hand, specific bioadhesives facilitate bioadhesion at the mucus layer or cell surface [14,19]. For instance, lectins are plant-derived proteins that can detect and specifically bind with a sugar molecule [20]. Bacterial adhesion to intestinal surfaces takes place because of the interaction between bacterial lectins and mucin, therefore lectins can act as bioadhesives [14]. Some other specific bioadhesive lectins include wheat germ agglutinin, fimbrin, and bacterial adhesins [21].

Bioadhesion can be grouped into three types in the case of biological systems. In type 1, adhesion occurs between two biological components, for instance wound healing and platelet aggregation [22]. In type 2, adhesion occurs between an artificial substrate and a biological component, for instance cell adhesion to culture dishes. In type 3, adhesion takes place between a biological substrate and an artificial material, for instance adhesion between soft tissues and synthetic hydrogels [17]. A number of polymer- and environment-associated factors have already been identified that can affect bioadhesion [23]. The polymer-associated factors include bonding of functional groups, attachment and bonding of functional groups, degree of hydration, chain flexibility, spatial conformation, concentration, and molecular weight [11]. In contrast, environment-associated factors include mucin turnover, initial contact time, pH, and the disease state of the mucus layer [24]. It has been observed that polymer-associated factors affect swelling, interpenetration, and adhesion strength, while environment-associated factors affect the force of adhesion and surface charge [6,25].

**Figure 2:**
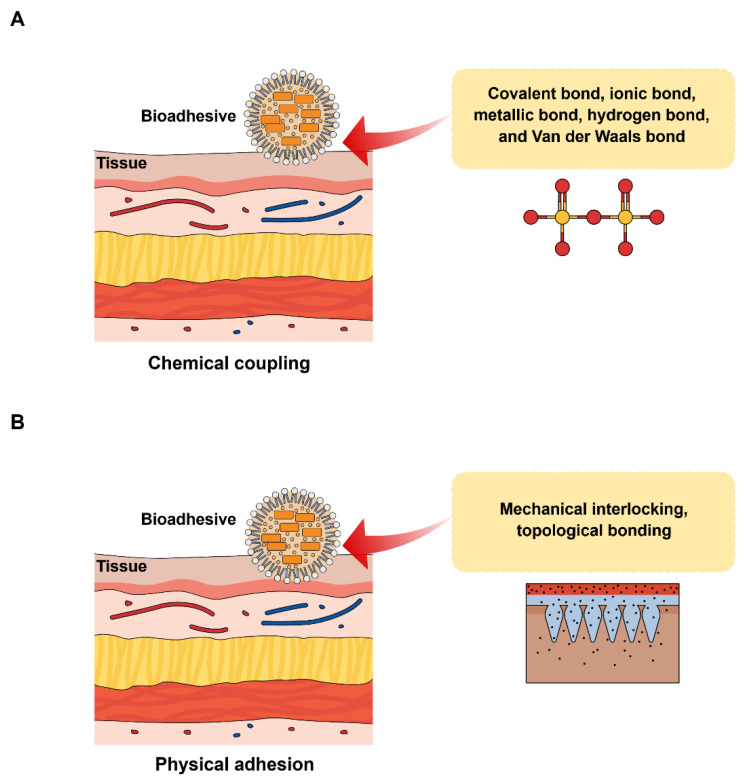
Mechanism of bioadhesives, (**A**) chemical coupling and (**B**) physical adhesion.

On the other hand, depending on the chemical and physical properties of tissues and bioadhesives, the mechanism of bioadhesives can be classified into chemical coupling and physical adhesion [15]. Figure 2 illustrates the different types of mechanisms of bioadhesives. In terms of chemical coupling at the nanoscale, the intermolecular interactions can be categorized into either primary (such as metallic, ionic, and covalent bonds) or secondary (such as Van der Waals bond and hydrogen bond), or a combination of both. In terms of physical adhesion, either topological bonding, mechanical interlocking, or both are present at the nanoscale. Researchers have already developed a range of natural and synthetic bioadhesives for their uses as potential drug delivery systems, which has been discussed in the next section.

## 4. Biopolymers Used to Prepare Bioadhesive Nanoparticles

### 4.1. Natural Biopolymers

#### 4.1.1. Gelatin

Gelatin is a protein that is widely used in biomedical, pharmaceutical (Table 1), and food industries owing to its unique biological properties, biocompatibility, and biodegradability. Gelatin is mainly obtained by physical degradation, chemical degradation, or thermal denaturation of collagen [26]. As compared to collagen, gelatin offers some advantages including modulable physicochemical properties, cost-effectiveness, and the fact that it is easier to obtain. Gelatin is recognized by the United States Food and Drug Administration (US FDA) as a safe material. However, gelatin shows poor adhesive and mechanical properties. Therefore, the drug-loading capacity, as well as the release kinetics of drugs from gelatin, are modified by its degree of crosslinking, regulating the gelatin-drug electrostatic interaction, and modifying the molecular weight of gelatin [21]. Gelatin is usually crosslinked to reduce the degradation rate of gelatin in vivo, to enhance hydration properties and mechanical stability under physiological conditions, and to prolong the release of drugs from gelatin-based delivery systems [21]. Crosslinking gelatin with various cross-linking agents including carbodiimide and genipin served as an excellent bioadhesive and showed substantially lower cytotoxicity [27]. Gelatin has been used as a structural component in a wide range of bioadhesive dosage forms including NPs, tablets, wafers, films, microparticles, microgels, and hydrogels. In a study, Ramirez-Barron et al. [28] developed the functionalization of gelatin with gallic acid, which further resulted in the generation of a crosslinked network with an organosilane in order to develop an efficient bioadhesive material. Then, the zinc oxide NPs were incorporated into the gelatin–catechol matrix. The obtained nanocomposites showed enhanced adhesion and antimicrobial properties, which have great potential in burn and wound dressings [28].

#### 4.1.2. Chitosan

Chitosan is a promising biopolymer derived from chitin [29]. Uses of chitosan have been extensively explored for numerous biomedical applications, including tissue engineering and drug delivery systems, because of its excellent absorption effect, bioadhesive properties, and biocompatibility [30]. Chitosan-based preparations have already shown enhanced wound-healing properties [31,32]. Several chitosan-based drug delivery systems have been studied, including hydrogels and NPs. Chitosan NPs show the same characteristics as chemically modified or natural polymers. Chitosan NPs are typically produced under mild conditions because of chitosan’s chemical properties. At room temperature, chitosan is soluble in acidic aqueous solutions, and no heat and toxic organic solvents are needed to prepare chitosan NPs [30]. Different types of drug molecules can be delivered by chitosan NPs, including polynucleotides, proteins, and small molecules. Chitosan NPs have been identified as a potential mode of transdermal, cutaneous, vaginal, periodontal, buccal, pulmonary, nasal, ocular, and drug delivery [30,33,34]. Furthermore, chitosan-based bioadhesives have extensively been used in the preparations of hemostatic and wound dressings. In a study, Dandamudi et al. [35] showed that chitosan-coated poly (lactic-co-glycolic acid) (PLGA) NPs showed enhanced thermal stability, which has great potential for topical ocular drug delivery against retinal vasculopathy.

#### 4.1.3. Collagen

Collagen is the most abundant protein found in the human body. It is widely found in ligaments, bones, tendons, and skin [10]. Collagen shows excellent biocompatibility, biodegradability, minimal immunogenicity, and effective bioadhesion. These features of collagen can be effective in wound healing and tissue regeneration [36]. It is also an effective, safe, and environmentally friendly source of nanocarriers as compared to other types of natural or synthetic polymeric NPs. Biodegradability and high cationic properties of collagen NPs-based wound dressings can mediate the local drug delivery to the wound via increasing absorption of the drug and by mediating the natural wound healing mechanism [36]. Collagen is also combined with various other polymers including chitosan and polyvinyl alcohol to ameliorate the generation of composite films as well as nanocomposites [37,38].

#### 4.1.4. Albumin

Albumins are globular proteins that are the most abundant serum albumins. Albumins have several advantageous intrinsic properties including nontoxicity, low immunogenicity, biodegradability, and biocompatibility. Commercially available albumins include human serum albumin (HSA) and bovine serum albumin (BSA) are commonly used in numerous biomedical and clinical applications [6]. Albumins have some unique capacities to bind with several compounds, therefore they have been used as a coating agent for biomaterial functionalization to decrease the non-specific adsorption of proteins and increase cell adhesion as well as the proliferation [39]. There are two common methods of preparing albumin-based drug delivery systems. One method involves the encapsulation of drugs in the albumin-based NPs, and the other method involves the generation of albumin–drug conjugates via the chemical coupling of drug molecules to single albumin molecules [39]. In terms of albumin-based bioadhesives, BioGlue is used along with traditional means to achieve hemostasis (including staples and sutures) in adult individuals in the open surgical repair of large vessels (including carotid arteries, femoral arteries, and aorta). The mechanism of this bioadhesion involves an aldehyde group from glutaraldehyde, which can interact with several functional groups, including amine groups, in the treated tissues and amine groups from BSA, which can eventually result in a strong bond between the tissue and BioGlue [40]. On the other hand, the only FDA-approved sealant, Progel Pleural Air Leak Sealant, contains a synthetic crosslinker PEG-(SS)_2_ and a human serum albumin (HSA) solution, which is clinically proven to seal air leaks in both open and minimally invasive lung surgery [41].

#### 4.1.5. Cellulose

Cellulose is a natural, biodegradable, non-toxic, biocompatible, and renewable biopolymer widely found in plants, bacteria, marine algae, tunicate, bagasse, sugarcane, and seaweeds [42]. Cellulose shows excellent physical, mechanical, and chemical properties, including high biodegradability, lightweight or low density, good elastic modulus, high tensile strength, chirality, and greater stability under acidic conditions [43,44]. Therefore, cellulose has been widely used in numerous biomedical applications, including tissue engineering, drug delivery, wound dressing, and the development of antibacterial agents [44,45,46]. It has been reported that bacterial cellulose can be utilized in case of burn injury to mediate cell migration as well as tissue regeneration [44]. There are several advantages of adding silver NPs (AgNPs) into bacterial cellulose-based wound dressings, including a reduction in the bacterial load on the site of burn injuries and the prevention of infection by suppressing bacterial growth [47]. In a study, Kovtun et al. [48] developed chlorhexidine-loaded calcium phosphate NPs for periodontal maintenance. They observed that carboxymethyl cellulose-coated NPs showed enhanced bioadhesion to dentin and enamel. Moreover, the BNPs were effective in suppressing bacterial growth [48].

### 4.2. Synthetic Biopolymers

#### 4.2.1. Poly(lactic-co-glycolic acid) (PLGA)

PLGA is an FDA-approved biodegradable synthetic polymer which is widely used in the delivery of various macromolecules, proteins, and drugs. PLGA is regarded as the gold standard of biodegradable polymers for controlled-release drug delivery systems [35]. In addition, PLGA-based bioadhesives are used extensively as bioadhesive systems. An example of a commercially available PLGA-based drug delivery system is Lupron Depot, which is used to treat advanced prostate cancer [49,50]. PLGA has already been used as a nanocarrier in several studies because of its high potential for drug delivery [51,52,53]. Chitosan can be used to coat PLGA-based nano-carriers to regulate drug release, wherein chitosan’s hydrophobic nature averts the elimination of nanocarriers by the reticuloendothelial system. Chitosans are cationic polysaccharides that contain amino and hydroxyl groups with the capacity to form covalent and hydrogen bonds. At low pH, the protonation of the amino groups leads to chitosan mucosal adhesion, which makes the chitosan-based nanocarrier a potential drug delivery system [12]. Chitosan binding with biological ligands (for example antibodies), chemical ligands, or targeting agents (for example folic acid) are effective methods of targeting nanocarriers. Folic acid has the capacity to bind precisely with folic acid receptors on the surfaces of cancer cells; therefore, folic acid-conjugated NPs can be efficiently utilized to target tumor cells [54].

#### 4.2.2. Poly(ethylene glycol) (PEG)

PEG is a non-toxic, biodegradable, non-ionic, synthetic, and hydrophilic polymer. PEG can also be modified to achieve bioadhesive characteristics in some drug delivery systems. PEG chains might establish explicit bioadhesive interactions with mucosal tissues owing to their capacity to interdiffuse across the mucus network [6]. In addition, it has been observed that PEG can ameliorate the targeting delivery capacity of NPs, which suppresses the elimination of NPs by the mononuclear phagocytic system and can also modify some physicochemical characteristics of NPs, including drug-release behavior, drug loading, stability, and membranes. It was observed that the density and molecular weight of PEG are the crucial factors that can affect the biological and physicochemical properties of NPs [55,56].

#### 4.2.3. Polyacrylic Acid (PAA)

PAA is a synthetic, biodegradable, biocompatible, water-soluble, and non-toxic polymer that shows bioadhesive properties which has been utilized in several hemostatic agents and drug-delivery carriers [57]. Polycarbophil is a weakly crosslinked PAA which can serve as a potential bioadhesive. PAA NPs have been widely investigated in various biomedical uses, including drug delivery systems, because of their unique ability to deliver proteins, genes, and drugs [57]. In a study, Chiang et al. [58] demonstrated that magnetic cisplatin-PAA nanocapsules can exert significant anticancer activity, along with negligible side effects and reduced toxicity, in mice with an A549 tumor. In another study, Lee et al. [59] evaluated the NP formulations of PAA-co-methyl methacrylate containing cisplatin, which showed enhanced antitumor properties in an animal tumor xenograft model.

**Table 1:** Potential biomedical and pharmaceutical applications of bioadhesive biopolymers.

Biopolymers	Type	Drug/Active Ingredient	Outcomes	References
Gelatin	Natural	Zinc oxide	Enhanced adhesion and antimicrobial properties	[28]
Chitosan	Natural	Methotrexate	Showed activity against the human cancer cells	[54]
Collagen	Natural	Curcumin	Rapid wound-healing	[37]
Albumin	Natural	Bovine serum albumin and genipin	Enhanced postoperative wound healing accompanied by residual tumors photothermal elimination	[60]
Poly(lactic-co-glycolic acid)	Synthetic	Silicon nitride	Mediated bone regeneration	[61]
Poly(ethylene glycol)	Synthetic	Tetra-poly (ethylene glycol) hydrogel	Mediated effective repairing of meniscus tears	[62]
Polyacrylic acid	Synthetic	Cisplatin	Enhanced anticancer activity with negligible side effects	[58]
Cellulose	Natural	Chlorhexidine	Enhanced bioadhesion to dentin and enamel	[48]

## 5. Applications of Bioadhesive Nanoparticles in the Treatment of Skin Disorders

**Figure 3:**
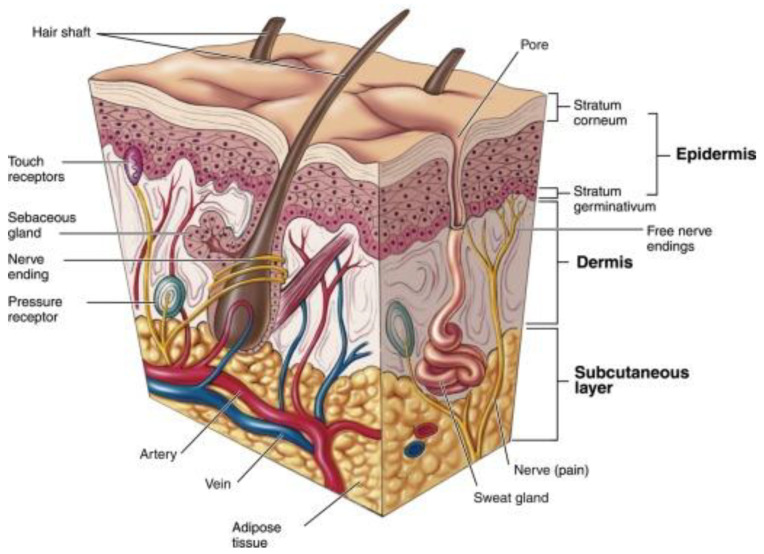
Representative image showing various skin layers. Reproduced with permission from Elsevier, Reference [66].

Skin is the largest organ of the human body, with unique anatomical and physiological features, which serves as the major functional barrier between the human body and the environment [63,64]. The three layers of the skin, including the epidermis, dermis, and hypodermis, cover the entire external surface of the human body (Figure 3). The epidermis plays an important role in barrier function, protection against UV radiation, and innate immunity. The dermis is the thickest layer of the skin, which is an integrated system of filamentous, fibrous, and amorphous connective tissues that accommodates a range of cell types including mast cells, macrophages, and fibroblasts. The dermis layer also contains nervous, lymphatic, and vascular networks. The subcutaneous layer provides both physiological as well as mechanical support, and serves a larger source of nerves and vessels [63].

Various skin problems or conditions can occur not only because of disorders of the skin itself, but also owing to several environmental hazards including pathogens, UV radiation, and physical as well as chemical injury [65]. Indeed, BNPs can serve as an efficient topical delivery system as they can serve dual purposes as bioadhesives and nanocarriers which can mediate targeted drug delivery, prolonged retention time, and deeper drug penetration through skin layers. A range of studies have already been carried out to evaluate the potential of BNPs in the treatment of various skin disorders, including atopic dermatitis (AD), irritant contact dermatitis, skin cancer, psoriasis, microbial infections, wounds, and severe burn injuries.

### 5.1. Atopic Dermatitis (AD)

AD is a very common inflammatory skin disorder, with around 10–20% occurrence in developed countries [67]. In addition, there is an increasing trend in the prevalence of AD worldwide. AD is commonly characterized by pruritus and recurrent eczematous lesions. AD hallmarks include inflammatory, relapsing, chronic, and pruritic skin disorder, which takes place because of the excessive infiltration of mast cells, immunoglobulin-E, and T lymphocytes. AD-related clinical manifestations can vary according to the severity of AD and the age of the patient [68]. AD pathogenesis is multifactorial and is caused by environmental triggers, genetic defects, weakened epidermal barrier integrity, and immune dysregulation [69]. Budesonide is a strong corticosteroid that exhibits strong anti-inflammatory activity and weak mineralocorticoid activity because of its low systemic toxicity. Budesonide can also suppress the activity of numerous cell types and inflammatory mediators. This strong corticosteroid is usually used as a topical therapy for AD [70]. Despite its strong anti-inflammatory properties, the prolonged use of budesonide can cause contact dermatitis in AD patients because AD patients often require the prolonged use of corticosteroids. Nano-based formulations have been widely investigated for the topical delivery of various drug types to enhance skin disease treatment [71]. Nanoencapsulation offers some benefits over traditional formulations, including enhanced apparent drug solubility, site-specific delivery, reduced side effects, reduced dose requirement for biological activities, and enhanced residence time of topical drugs in the dermis, epidermis, and stratum corneum. Moreover, nanoencapsulation reduces systemic toxicity and enhances bioavailability [72,73].

Because of the hydrophobic nature of PLGA, it can be used to load various hydrophobic drugs, including budesonide [74]. In addition, coating PLGA NPs with chitosan was found to enhance the sustained release property [75]. Chitosan is a cationic polymer that facilitates their interaction with the cell membrane and also provides excellent biodegradability, bioadhesion, and formability [76]. Therefore, a stimuli-responsive system containing sustained release and bioadhesive properties can be developed by incorporating the aforementioned biopolymers [77]. In a study, Campos et al. [70] encapsulated budesonide into chitosan-coated PLGA NPs, which were further incorporated into poloxamer hydrogels in order to reduce adverse effects and enhance anti-inflammatory activity. The NPs in this study were prepared by using the emulsification–solvent evaporation method [77]. The developed NPs had a mean diameter of 324 ± 4 nm, high encapsulation efficiency (over 90%), and a positive zeta potential (20 mV) because of the cationic polymer chitosan coating. The researchers summarized that the NPs did not exert cytotoxic activities in primary human fibroblasts and keratinocytes. Moreover, the developed nanoencapsulation mediated the skin absorption of budesonide, and the nano-formulations may play a role as a potential candidate in delivering glucocorticoids in the skin of patients with AD [77].

### 5.2. Irritant Contact Dermatitis (ICD)

ICD is an inflammatory skin disease that occurs in exposure to a highly irritating agent, which can further result in the activation of the innate immune system. ICD can take place owing to skin barrier damage caused by environmental factors or external agents. This skin disorder typically affects hands and can affect people of all ages, which is more common than allergic contact dermatitis [78]. Certain occupations are at greater risk of developing ICD, including construction workers, hairdressers, metal workers, food service workers, and healthcare workers [79]. ICD clinical manifestations include stinging and burning that typically starts within a few seconds after exposure [80]. Current ICD treatments include topical corticosteroids and emollients. However, the prolonged use of topical corticosteroids is avoided because of the alteration of the dermal barrier, reduced regeneration of skin barrier, and induction of cell atrophy [81]. Polymeric NP-based dermal delivery systems can be effective in the treatment of various skin disorders because polymeric NPs can enhance the skin permeation capacity of drugs, particularly in poorly water-soluble lipophilic drugs. This enhancement is attained by elevating the drug concentration gradient across the skin, which results in enhanced drug stability, reduced side effects including skin irritation, and site-specific drug delivery to minimize systemic exposure [81]. Hyaluronic acid and chitosan are biopolymers that can be used to generate polymeric NPs because of their excellent nontoxic properties, biodegradability, and biocompatibility. Indeed, chitosan shows excellent skin retention time through bioadhesion because of the polyanionic regions on the skin surface and the cationic nature of chitosan [82].

Hyaluronic acid is a naturally occurring anionic biopolymer, which mediates skin barrier repairing process [83]. Hyaluronic acid is extensively used in dermal drug delivery in order to improve drug penetration. In addition, this anionic biopolymer shows good solubility properties [84]. Etoricoxib is a potent non-steroidal anti-inflammatory drug, which shows delayed release, delayed onset of action, poor dissolution rate, and low water solubility which hinders its uses in topical delivery [85]. In a study, Abuelella et al. [86] fabricated polyelectrolyte complex NPs (PENPs) containing hyaluronic acid and chitosan in order to deliver etoricoxib in the deeper layer of the skin to improve its therapeutic efficacy against ICD and to reduce systemic toxicity caused by etoricoxib. The researchers observed that the PENPs markedly ameliorated the etoricoxib deposition in the dermal, epidermal, and subcutaneous layers as compared to the conventional etoricoxib gel. This enhanced etoricoxib deposition mediated by the PENPs was attributed to the bioadhesive properties of hyaluronic acid and chitosan, which mediated the attachment of PENPs with the skin appendages and enhanced the well-controlled discharge of their active ingredients locally [86]. Moreover, etoricoxib-loaded PENPs showed enhanced in vivo anti-inflammatory properties in comparison with the conventional etoricoxib gel in a mouse model. The researchers also concluded that etoricoxib-loaded PENPs can be a novel and effective approach in the treatment of ICD [86].

### 5.3. Skin Cancer

Skin cancer is one of the most dangerous forms of cancer, and is the fifth most common type of cancer [87]. In 2018, around 9.6 million skin cancer-associated deaths and 18.1 million new cases were reported worldwide [88]. Moreover, it has been estimated that the number of new cases of skin cancer is likely to increase over the next 20 years [89]. The etiology of skin cancer lies in the abnormal proliferation of skin cells mediated by the unrepaired DNA damage to skin cells, which triggers mutations or genetic defects. Cancer cells that arise from mutations in skin melanocytes are known as malignant melanoma [90]. The most common type of skin cancer is non-melanoma skin cancer, which starts in the epidermis layer of the skin [88,91]. It is now widely known that cumulative UV radiation exposure from sunlight is the major risk factor for skin cancer, which can result in UV-mediated changes in the expressions of skin proteins [92]. UV radiation exposure is known as a complete carcinogen, as it can affect each step of carcinogenesis. Furthermore, it results in cellular injury owing to DNA alteration, the generation of reactive oxygen species, and decreased cell-mediated immune responses [93]. Currently, available topical drugs for the treatment of skin cancers include photodynamic therapy, cryotherapy, or topical chemotherapeutics. However, the use of these therapies is limited because of various problems including light sensitivity, complexity, and cost. Moreover, these therapies are only useful in the treatment of superficial skin lesions and often fail to reach the deeper layers, which leads to the emergence of newer lesions or progression to distant regions [94].

The use of nanotechnology approaches has great potential to improve the therapeutic techniques used in skin cancer treatment. BNPs have a therapeutic promise in improving the selectivity of targeting cancer cells by mediating the targeted delivery of drugs specifically to tumor cells, because of their enhanced retention effect and permeability [87]. As compared to conventional chemotherapies, NPs generated from polylactic acid–hyperbranched polyglycerol (PLA-HPG) copolymers can improve the efficacy, bioavailability, and duration of topically delivered chemotherapy drugs, along with a reduced systemic toxicity. Brief incubation with sodium periodate is required to convert non-adhesive PLA-HPG NPs to BNPs [95]. The generated BNPs have the capacity to efficiently bind through the covalent bonds with amine groups present in tumor interstitial matrix proteins and on the tumor cell surfaces. It has been reported that the bioadhesive effect exerted by these NPs can prolong the retention time of BNPs with intracranial tumor cells, vaginal epithelium, mesenteric membranes, and stratum corneum [95,96,97]. Camptothecin is a natural plant alkaloid and anticancer drug which shows limited therapeutic efficacy because of systemic adverse effects, rapid physiological inactivation, and poor water solubility [98]. To overcome these limitations, Hu et al. [99] devised a BNP-based topical delivery system containing PLA-HPG-encapsulating camptothecin. Their generated BNPs showed improved binding with the squamous cell carcinoma tumors and matrix proteins, which substantially increased the therapeutic effectiveness of intratumoral drug delivery. Moreover, they concluded that the percutaneous delivery of these BNPs might be a potential nonsurgical alternative for the treatment of skin cancer [99].

### 5.4. Psoriasis

Psoriasis is a common, immune-mediated inflammatory skin disorder which has a profound physical and mental health comorbidity [100,101]. Psoriasis affects nearly 2–5% of the global population, and its common clinical manifestations include psoriasis pustulosa, psoriasis erythrodermic, psoriasis arthropathica, and psoriasis vulgaris. Among them, psoriasis vulgaris is the most common form of psoriasis, which is characterized by scaly, erythematous skin lesions containing distinct borders [102]. Psoriasis pathogenesis includes immune response irregularities (for example, adaptive immune system overactivation), various environmental triggers (for example, smoking, alcohol consumption, obesity, stress, and streptococcal infections), and genetic predisposition. Conventionally, topical treatment is regarded as the primary option to treat individuals with mild-to-moderate plaque psoriasis, while maintenance topical treatments are used in individuals with moderate-to-severe psoriasis to defer relapse [103].

Fucoxanthin is a marine carotenoid that has been found to be useful against various diseases and dysfunctions, including inflammation-related disorders, hypertension, cancer, diabetes, heart disease, obesity, and metabolic syndrome [104]. Fucoxanthin-rich seaweeds have traditionally been utilized in Southeast Asia as remedies for digestion, improved reproduction, skin rejuvenation, and blood purification [104]. Fucoxanthin also exerts antiproliferative activities, which can be effective against hyperproliferative conditions including psoriasis [103]. In a study, Cordenonsi et al. [105] used nanostructured lipidic carriers (NLCs) by using tucuma oil and bacuri butter loaded with fucoxanthin for topical application to avert hyperproliferative conditions in the skin including psoriasis. They also used chitosan to coat the NLCs to ameliorate their bioadhesion and wound-healing properties. The average particle size range of the NLCs was between 250 and 400 nm. It was observed that chitosan-coated, fucoxanthin-loaded NLCs did not exert any toxic effect and significantly reduced the skin inflammation and hyperproliferation to preserve skin integrity in psoriatic skin [105].

### 5.5. Bacterial Skin Infections

Bacterial skin infections, such as cellulitis and pyoderma, involve a substantial global burden of disease. Antibiotics are primarily used in the treatment of bacterial skin infections. Topical antibacterials are typically utilized to treat superficial pyodermas including impetigo and in the prevention or treatment of infections following surgical wounds, burns, abrasions, and minor cuts [106]. There are several advantages of using topical antibacterials in the treatment of skin infections including targeted drug delivery, excellent bioavailability, no impact on beneficial intestinal microbiota, and low cost. Unfortunately, the effectiveness of antibiotics is decreasing because of the existence of antibiotic-resistant bacterial strains [107]. Thus, numerous NP-based delivery systems have been developed to enhance therapeutic action and overcome antibiotic resistance by altering their biodistribution profile and pharmacokinetics, such systems include various inorganic NPs, dendrimers, polymeric NPs, and liposomes [108]. In order to improve topical antimicrobial drug delivery, Zhang et al. [109] developed a bioadhesive NP gel system in their study. In the formulation, ciprofloxacin (a broad-spectrum antibiotic) was encapsulated by PLGA for sustained and controlled release. It was observed that the developed bioadhesive NP gel exhibited enhanced antibiotic retention and adhesion on mouse skin, and also suppressed the generation of *Escherichia coli* bacterial film, under a flow environment. Moreover, the bioadhesive NP gel containing ciprofloxacin did not cause any noticeable skin reaction or toxicity, which suggests its potential as a platform against microbial infections [109].

### 5.6. Wounds

The management of wounds is a major challenge globally, which involves an enormous financial burden [110,111]. Wound dressings are used to cover wounds to provide protection against further damage and mediate wound healing [110]. Furthermore, wound dressings play a role as an impermanent skin alternate and have significant contributions in wound closure, infection control, and hemostasis. Despite numerous available wound dressings, still there are some unmet needs. For example, most of the currently available wound dressings cannot adapt to the changing conditions of the wound [112]. In addition, pain caused due to the wound dressing changes is another issue. Treating skin ulcers in certain areas with currently available dressings is challenging compared to treating flat areas because of the inadequate coverage of the wound site, poor adhesion performance, difficulty fixing, and unavoidable movements [113]. In order to overcome the aforementioned limitations, an increasing number of studies are focusing on the development of robust bioadhesive nanocomposite hydrogels because of their excellent antimicrobial properties, elasticity, and mechanical strength [114]. There are a range of processes that are linked with wound healing that include a delicate balance between antioxidants and oxidative stress, where an elevated level of oxidative stress might impair the wound healing process, particularly when comorbidities (for examples diabetes) are present [115,116]. Therefore, treatment with antioxidants was found to control wound-associated oxidative stress and mediate healing [117]. In a clinical study, as compared to hyaluronic acid, treatment with hydrogels containing antioxidants oleic acid and quercetin reduced the healing time of lower limb wounds of patients with diabetes [118].

In a study, Costa-Fernandez et al. [119] developed NLCs to provide both antimicrobial (tea tree oil) and antioxidant (quercetin and α-tocopherol) properties for wound management. NLCs were prepared using argan oil and shea butter, which were further modified by using chitosan or sodium alginate to mediate bioadhesive properties. They obtained spherical NPs with a zeta potential ranging between −21.2 and +11.8 mV and particle size of ~307–330 nm. It was observed that the antioxidants present in the NLCs enhanced NLC-mediated fibroblast migration, which suggests their potential use in wound management [119]. Bimetallic NPs are prepared by combining two different metals which provide a synergistic effect in comparison with the individual metals. It is well-known that gold (Au), Ag, and copper (Cu) are capable of exerting significant therapeutic effects. Thus, stronger therapeutic action can be achieved by combining these monometallic coinage metals into bimetallic NPs [120]. So far, AgNPs have extensively been studied for their strong antibacterial properties [121]. *Staphylococcus aureus* is a Gram-positive bacterium and a major human pathogen. Globally, *S. aureus* is the most common type of bacteria responsible for skin infections, regardless of geographical area, climate, and age of the patient [122]. Unfortunately, *S. aureus* has the capacity to develop resistance against nearly all classes of clinically available antibiotics [123]. The chitosan-encapsulated bimetallic NPs have been identified as a potential therapeutic to fight against microbial infections [124].

Singh et al. [125] developed chitosan-capsulated gold–silver bimetallic NPs (CS-AuAg-NPs) by using a seeded growth synthesis method. The mean particle size of CS-AuAg-NPs was between 55 and 289 nm, and the zeta potential ranged between +8.53 mV and +38.6 mV. It was observed that, compared to the clindamycin standard, CS-AuAg-NPs showed greater efficacy against multidrug resistant (MDR) *S. aureus* ATCC 25923 and MDR 1695. Moreover, CS-AuAg-NPs distributed in the chitosan film mediated 87% of wound recovery after one week of treatment in a mouse model. Mussels exhibit a remarkable adhesive property; therefore, they are harnessed for various bio-inspired uses, including bio-medical adhesive materials. Mussels secrete a type of liquid protein which is known as a nature’s glue that rapidly hardens into a solid water-resistant adhesive substance [126]. The incredible adhesion property of mussels is credited to amino acid 3,4-dihydroxy-L-phenylalanine (L-DOPA)-containing proteins. The catechol moiety of L-DOPA further enhances the adhesion [126]. Gallic acid is a natural polyphenol, which is widely found in various plants. This natural polyphenol shows antioxidant, anti-inflammatory, antiviral, and antibacterial properties. In addition, gallic acid shows antimelanogenic activity by suppressing tyrosinase activity [127]. Like L-DOPA, gallic acid can strongly bind with titanium (IV), iron (III), and various other metals. The metal ions have the capacity to displace protons from the -OH group, which can result in the formation of an inner sphere complex. Because of the capacity of gallic acid to bind with various metals and substrates, it is considered as a potential bioadhesive. Glycidoxy-propyl-trimethoxi-silane (GPTMS) is a biodegradable and biocompatible organosilane which is used as a crosslinker of various biomaterials and in the enhancement of bioadhesive properties [128].

Zinc oxide NPs (ZnO NPs) have already been identified as potential antimicrobial agents. The antimicrobial activities of ZnO NPs are attributed to the generation of reactive oxygen species and electrostatic interaction between the microbial cell-surface and NPs, which eventually results in photodestruction through oxidative stress [129]. Indeed, ZnO NPs are capable of suppressing pathogenic microbial growth at very low concentrations, which is advantageous as compared to other types of NPs [130]. Ramirez-Barron et al. [28] functionalized gelatin with gallic acid to produce a cross-linked network with GPTMS in order to generate an effective bioadhesive. As antimicrobials, ZnO NPs were loaded into the gelatin–catechol matrix. It was observed that the developed nanocomposites showed enhanced antimicrobial properties, particularly at 3% wt of ZnO NPs. The researchers also concluded that the developed nanocomposite material showed enhanced antimicrobial and superior adhesive properties, which can serve as a potential application for burns and wounds [28].

### 5.7. Severe Burn Injuries

The mortality rates of burn victims are comparable to the mortality rates of acute myocardial infarction [131]. Globally, burns are considered as the fourth major cause of trauma, which affects over 11 million people every year [132]. Among them, more than 90% of burn injuries are reported in middle- to low-income countries [133]. In general, around 5% mortality rates are observed in the case of severe burns, where most of them are associated with sepsis, infection, multiple organ failure, immune compromise, and hyperinflammation. Furthermore, poor outcomes are often linked with the presence of polytrauma, preexisting comorbidities, advanced age, and burn severity [134]. Although mortality rates have decreased over the last few decades, the optimal resuscitative regimens and treatment are yet to be developed to treat severe burn injuries, which are more important when burns occur along with hemorrhage or any other trauma [134,135]. Autologous skin grafting and early wound debridement are currently considered as effective methods of repairing wounds. Nonetheless, patients with severe burn injuries in some cases cannot provide adequate autografts [136]. Henceforth, skin allograft is widely used to treat patients with severe burns. Unfortunately, graft rejection is a major challenge in the case of allografts [137]. Because of the high risk of systemic toxicity, no specific immunosuppressive agent is currently suggested for skin allografts [137].

A number of immunosuppressive approaches including natural ingredient-based therapy, antibody therapy, and stem cell therapy have been investigated as a potential method to minimize adverse effects and optimize efficacy [138]. Nevertheless, immune rejection hindered the healing mediating activities of allograft skin, which is a major challenge in the case of allogeneic skin grafts [139,140]. Rapamycin is a strong immunosuppressant that exhibited minor side effects in various studies [139]. It has been observed to hinder allograft rejection in obese graft patients, which was found to be linked with its capacity to suppress the immune system and exert anti-inflammatory activity within subsets of immune cells. In addition, the co-delivery of programmed death-ligand 1 and rapamycin within nanovesicles can achieve allograft acceptance [141]. In a study, Liu et al. [139] developed a BNP-based topical delivery system containing PLA-HPG encapsulating rapamycin. In a mouse model of allogeneic skin grafts, the topical formulation was locally applied for immunosuppression. The developed BNPs markedly reduced the level of proinflammatory cytokines, reduced the infiltration of macrophages and T lymphocytes, and extended the retention of NPs, which eventually resulted in extended skin allograft survival along with slight systemic toxicity in comparison with the rapamycin alone or non-bioadhesive NPs loaded with rapamycin [139]. Table 2 summarizes the applications of bioadhesive nanoparticles in skin disorder treatments.

**Table 2:** Potential applications of bioadhesive nanoparticles in the treatment of skin disorders.

Skin Disorders	Bioadhesive Nanoparticles	Drug/Active Ingredient	Outcomes	References
Atopic dermatitis	Chitosan-coated poly (lactic-co-glycolic acid) nanoparticles (NPs)	Budesonide	Mediated the skin absorption of budesonide; did not exert cytotoxic activities in primary human fibroblasts and keratinocytes	[77]
Irritant contact dermatitis	Polyelectrolyte complex NPs (PENPs) containing hyaluronic acid and chitosan	Etoricoxib	Etoricoxib-loaded PENPs showed enhanced in vivo anti-inflammatory properties in comparison with the conventional etoricoxib gel	[86]
Skin cancer	Bioadhesive NPs (BNPs) containing polylactic acid-hyperbranched polyglycerol (PLA-HPG) copolymers	Camptothecin	Camptothecin showed increased therapeutic effectiveness	[99]
Psoriasis	Chitosan-coated nanostructured lipidic carriers (NLCs)	Fucoxanthin	Fucoxanthin-loaded NLCs did not exert any toxic effect and significantly reduced skin inflammation and hyperproliferation to preserve skin integrity in psoriatic skin	[105]
Bacterial skin infections	Poly(lactic-co-glycolic acid)-based BNP–hydrogel hybrid	Ciprofloxacin	BNP gel enhanced antibiotic retention and adhesion on skin; suppressed the generation of *Escherichia coli* bacterial film	[109]
Wound	Gelatin–gallic acid/zinc oxide NPs	Zinc oxide	Enhanced antimicrobial and superior adhesive properties	[28]
Severe burn injuries	BNP based on PLA-HPG	Rapamycin	Extended skin allograft survival with slight systemic toxicity	[139]

## 6. Challenges and Future Directions

There is an increasing demand for BNP-based applications in medicine because of the various advantages, including targeted or specific drug delivery and slow, as well as prolonged, residence time at the site of drug application. Furthermore, BNPs show a similar or higher strength compared to commercial glues. As most of the BNPs are biocompatible, their use can prove beneficial in clinical settings. Several studies are currently ongoing in the development of BNPs to meet the requirements of degradability, flexibility, biocompatibility, and enhanced adhesive strength. The current progress of these studies is promising an enhanced capacity of BNPs to mediate improved clinical outcomes. Lectin-based bioadhesives have been reported as effective in selectively targeting cells by binding with certain sugar groups. Therefore, lectin-based BNPs might achieve specific-targeted delivery of drugs as a second-generation bioadhesive. The combination of bioadhesives with various other delivery systems might be effective to mediate efficient drug delivery and prolong drug release on skin. Nonetheless, bioadhesives are not free from drawbacks. Natural material-based bioadhesives show excellent biodegradability and biocompatibility, but they have proinflammatory potential, lack bulk strength, are difficult to store, and are expensive. In contrast, bioadhesives based on synthetic materials show low immunogenicity, a controllable structure, and excellent mechanical properties, Nonetheless, most of the synthetic materials show weak bioactivity and biofunctionality; therefore, they need to go through required postprocessing to mediate the desired effects [142]. On the other hand, currently available bioadhesives, including commercial FDA-approved bioadhesives, have several drawbacks, including poor interfacial bonding with biological tissues, slow adhesion formation, and cytotoxicity [14]. It is more expensive to produce bioadhesives than their synthetic counterparts. In addition, all the newly developed bioadhesives need approval before commercialization, which can be expensive and time-consuming. Therefore, newly developed bioadhesives should be biocompatible and should contain the required properties that are set by the standard guidelines, which will eventually accelerate the regulatory approval process.

## 7. Conclusions

There is an increasing demand for multifunctional and new delivery systems based on bioadhesion in combination with various nanotechnologies. BNPs can serve as an effective topical delivery system as they can serve dual purposes as bioadhesives and nanocarriers. In terms of topical drug delivery, the encapsulation of various active ingredients into BNPs provides unique benefits compared to free drugs. BNPs can also be improved further by combining them with various other advanced therapies. Significant progress has been achieved in the preparation of several effective BNPs, however the various drawbacks associated with BNPs are yet to be fully addressed, such as weak bonding, slow adhesion formation, proinflammatory potentials, and batch-to-batch variability. Henceforth, rigorous research along with clinical studies are required to improve knowledge regarding BNPs for their potential application in topical drug delivery.

## Data Availability

The data presented in this study are contained within this article.
